# A visualization pipeline for *in vivo* two-photon volumetric astrocytic calcium imaging

**DOI:** 10.7555/JBR.36.20220099

**Published:** 2022-08-10

**Authors:** Qian Sun, Yusi Hu, Saiyue Deng, Yanyu Xiong, Zhili Huang

**Affiliations:** 1 Department of Pharmacology, School of Basic Medical Science, Fudan University, Shanghai 200032, China; 2 State Key Laboratory of Medical Neurobiology, Institutes of Brain Science and Collaborative Innovation Center for Brain Science, Fudan University, Shanghai 200032, China; 3 Department of Neurology, Tongji Hospital, Tongji Medical College, Huazhong University of Science and Technology, Wuhan, Hubei 430030, China

**Keywords:** astrocyte, calcium imaging, three-dimensional visualization

## Abstract

Astrocytes, the multi-functional glial cells with the most abundant population in the brain, integrate information across their territories to regulate neuronal synaptic and cerebrovascular activities. Astrocytic calcium (Ca^2+^) signaling is the major readout of cellular functional state of astrocytes. The conventional two-photon *in vivo* imaging usually focuses on a single horizontal focal plane to capture the astrocytic Ca^2+^ signals, which leaves >80% spatial information undetected. To fully probe the Ca^2+^ activity across the whole astrocytic territory, we developed a pipeline for imaging and visualizing volumetric astrocytic Ca^2+^ time-lapse images. With the pipeline, we discovered a new signal distribution pattern from three-dimensional (3D) astrocytic Ca^2+^ imaging data of mice under isoflurane anesthetic states. The tools developed in this study enable a better understanding of the spatiotemporal patterns of astrocytic activity in 3D space.

## Introduction

Astrocytes are highly diversified cells abundant in the central nervous system, with an individual cell expanding to 40 to 60 μm in diameter^[1–2]^. Astrocytes communicate with neurons in the form of "tripartite synapses", where the processes of the astrocyte warp around neuronal synapses^[3]^. Meanwhile, the endfeet of the astrocytes shape a shell around blood vessels, forming an essential component of the blood brain barrier^[4]^. This topological feature enables astrocytes to integrate distinct inputs arising from both neurons and the microcirculation^[5]^. As a major readout of astrocyte activities, astrocytic Ca^2+^ signaling has been certified to be involved in functions ranging from synaptic transmission, neural activity to blood flow regulation^[6–10]^, revealing that astrocytes play a fundamental part in the maintenance and regulation of neuronal networks. On the other hand, astrocytic activities are initialized by various subcortical ascending signaling, implying the active role of astrocytes in brain state modulation^[11–13]^.

Elevated intracellular calcium signaling in astrocytes has been observed in scope of spatial scales^[14]^. When in a smaller scale, the astrocytic processes demonstrate frequent asynchronously transient activities, while in a larger scale, the somatic activity often expands to the whole cellular territory with duration of tens of seconds. Heterogenicity is another characteristic. Even within an individual process, Ca^2+^ activity is complex and comprises different types of events^[8,15]^ owing to the activation of a series of functional subdomains. Beyond the scope of a single cell, astrocytes form syncytium by interacting with adjacent astrocytes *via* gap junctions^[16]^. Gap junctions synchronize groups of astrocytes by providing channels for Ca^2+^ to move intercellularly^[17]^. Astrocytes also interact with other cells by gliotransmission. Gliotransmitters, such as glutamate, ATP, and adenosine, are released by astrocytes in a Ca^2+^-dependent manner to mediate the interactions with other glial, neuronal or vascular cells^[18]^. In whole-brain scale, it has been proposed that astrocytes control extracellular ion concentration globally, especially potassium ion (K^+^), for potential synchronization across the entire networks and brain regions^[19]^. Accumulating evidence points to the hypothesis that the multiple-layered function by astrocyte Ca^2+^ signaling is ideally suited for modulating the neuronal network dynamics with a complex spatial structure^[20]^. As expected, several studies have revealed the roles of astrocyte Ca^2+^ signaling in various brain states, such as sleep, wakefulness^[21–22]^ and anesthesia^[23]^. Thus, probing into the input integration and output regulation with higher complexity and heterogeneity by astrocytes is crucial to the understanding of the mechanism of brain state transition. However, the mainstream astrocytic functional studies utilized two-dimensional (2D) imaging in a single focal plane, leaving a large proportion of spatial information undetected.

The study by Volterra's team has proved that 3D scanning can extend the enrichment of information from Ca^2+^ manifestations in a third dimension (the z-axis)^[24]^. To explore the role of astrocytic Ca^2+^ signaling in various brain states in 3D, we expressed astrocyte-specific highly sensitive calcium indicator, GCaMP6s, and injected SR101 as morphological labeling in the sensory cortex of mice. The cortical astrocytes at 100 to 150 µm below the surface was scanned with XYZT paradigm by a two-photon microscope during wakefulness and anesthetic condition. Meanwhile, we established a pipeline for visualizing 3D volumetric time-lapse images. We found different levels of activities and signal hotspots distribution in astrocytic Ca^2+^ dynamics between the states. This study provided a tool towards a better understanding of the spatiotemporal patterns of astrocytic activity, as well as the role of astrocytes in modulating neuronal network and brain states.

## Materials and methods

### Animals

Male C57BL/6J mice weighting 20 to 26 g (8 to 10 weeks old) were obtained from the Laboratory Animal Center, Chinese Academy of Sciences (Shanghai, China). All mice were group-housed (3 to 5 per cage) under a constant temperature ([22±0.5] °C), humidity ([55±5]%), and an automatically controlled 12-hour light/12-hour dark cycle (lights on at 7 a.m., illumination intensity approximately 100 lux), with access to food and water *ad libitum*. All experimental procedures were approved by the Animal Care and Use Committee of Fudan University (Approval No. 2020-0032). Every effort was made to minimize the number of animals used and any pain and discomfort experienced by the animals.

### Virus injection, astrocyte morphological labeling and chronic window preparation

Mice were anesthetized with isoflurane (2% for induction, 1% for maintenance) and placed in a stereotaxic frame (RWD Life Science, China). A heating pad was used to keep the constant temperature throughout the operation. After skin disinfection with 75% ethanol, the scalp was incised and the periosteum was removed from the skull. A circular craniotomy (4 mm in diameter) was made over the primary somatosensory cortex *via* a dental drill. Then a pipette and air pump were used to inject AAV2/5-gfaABC1D-GCamp6s virus (BrainVTA, China; 300 nL of 1×10^13^ V.G./mL at 60 nL/minute) into the target region (AP: −1.5 mm; ML: −2 mm; DV: −0.3 mm). The pipette remained in the injection site for 10 minutes and then was pulled out slowly. The surface of the skull was rinsed by sterile saline, and the exposed cortex was sealed with a 4 mm glass coverslip subsequently. The gap between the edge of coverslip and skull was filled with medical glue, and strengthened by dental cement. A customized head-fixing stainless-steel sheet was attached to the skull with dental cement mixed with super glue. Animals were given flunixin meglumine injection (0.02 mL/kg) for 3 days following the surgery and allowed to recover for at least 2 weeks before two-photon imaging. Astrocyte morphology was labeled by SR101 (HY-101878, MedChemExpress, China) 1 hour before imaging session. Intravenous injection of SR101 solution (0.3 mL) dissolved in saline (5 mmol/L) was conducted through the tail vein.

### Animal head-fixing training for two-photon imaging

At 3 to 4 weeks after the chronic window preparation, mice were fixed to an imaging frame to adapt to the later *in vivo* imaging sessions. Each training process lasted for 30 minutes/day, and for 5 days in total. The training process helped to reduce the stress with head restrain^[25]^.

### *In vivo* two-photon imaging

After the 5-day conditioning, the head-fixed awake mice were immobilized in homemade tubes to prevent wild free movement, and imaged under the Olympus FluoView FVMPE-RS upright multiphoton laser-scanning system with an Olympus XL Plan N 25×/1.05 WMP ∞/0–0.23/FN/18 water immersion objective lens (Japan). Both GCamp6s and SR101 were excited at 920nm with an INSIGHT X3-OL laser (Spectra-Physics, USA). Laser power was kept below 30 mW to avoid phototoxicity. Emitted fluorescence was detected through 495 to 540 nm and 575 to 645 nm bandpass filters. For 3D astrocyte calcium signal imaging, the calcium transients were recorded in cortical layer 2/3 100 to 300 µm below the pial surface. Appropriate cells were selected for imaging based on SR101 by adjusting zoom factor and z-axis. Image volume was scanned at 512×512 pixels for 20 to 30 layers in 2 μm z-axis step, resulting 0.121 to 0.144 µm lateral resolution and 2 µm axial resolution. Each plane was scanned twice and averaged for noise cancelling consideration. The volumetric sampling rate was 0.25 to 0.33 Hz. Typically, acquisitions of 2 to 3 minutes were made for each field of view. Since all mice received aseptic chronic cranial window surgery, imaging multiple trials in weeks was feasible.

### Isoflurane anesthesia during two-photon imaging

To compare the pattern of 3D calcium signaling in the same astrocytes under wakefulness and anesthesia, the mouse was first imaged for 20 minutes under the normal unstimulated condition, followed by the inhalation of 1.5% isoflurane through a nose cone for 40 minutes, and withdrawal of isoflurane for another 30 minutes. At each stage, the same astrocytes were imaged for 2- to 3-minute trial, with 1 minute interval for the first 15 to 20 minutes. During the rest of the session, imaging trial was acquired every 5 minutes.

### Volumetric image preprocessing

We performed XY-plane registration for motion correction. Image stack files (Olympus multi-photon imaging file format *.OIR or *.TIF) were imported into Fiji *via* bio-format importer plugin. Fiji plugin 'HyperStackReg' (https://github.com/ved-sharma/HyperStackReg) was utilized for the layer-wise shift correction with SR101 channel as reference. 'Rigid Body' method was chosen to maintain each layer proportional to the original.

### Volumetric image time projection and visualization

Image stack files (*.OIR or *.TIF) were imported into IMARIS 9.0 (Bitplane AG, Switzerland) *via* Imaris Format Converter. To obtain the average-over-time view of calcium signals, 'Image Processing'-'Time Projection' command was executed to create a signal heatmap (MATLAB XTension addon should be enabled in IMARIS, https://imaris.oxinst.com/open/). Contrast and brightness were kept original for cross-image comparison. The camera type was set as 'Orthogonal' for an unbiased 3D view. 'Gaussian smooth' with 1-pixel radius was used for visualization only. After choosing a desired angle of view, 'Snapshot' function was used to capture a 2D photograph of the volume. To view the XZ-XY-YZ orthogonal section, 'Slice'-'Section' view was enabled. Transparent background color was set under 'Preferences'-'Snapshot'.

### 3D surface reconstruction

A surface was reconstructed for labeling or masking of spatial region of interest, which was essential for further regional signal analysis. Imaris provided a semi-manual reconstruction tool. 'Add new Surface' Panel was then activated to initialize surface reconstruction. Under the parameter tuning step, 'Absolute value' was adopted as the thresholding method. The threshold value was set to lower quantile. Seeding was enabled with a size of 1 pixel. After reconstruction, the different parts from seeding method were combined by 'Unify' command.

### 3D image mask construction

Volume Segmenter App, an alternative 3D mask reconstruction tool was provided by Matlab Computer Vision Toolbox (Mathworks, USA). The app generated a 3D categorical-valued mask directed into the Matlab workspace with the same dimension as the input image stack. The mask stored as a Matlab variable was particularly useful when the signal in masked region needed to be processed by a customized script. To start, *.OIR or *.TIF files were imported into Matlab workspace *via* Bio-formats plugin (https://docs.openmicroscopy.org/bio-formats/6.1.0/users/matlab/index.html). Volume Segmenter App was under 'Apps' Tab, 'Computer Vision' Section (Matlab R2021a onwards). The image stack could either be imported from a file or from a Matlab n-by-m-by-p matrix. The mask construction was a 3-step process. First, areas of interest in each plane (z-axis) were manually drawn with the borders finely tuned by 'Erode' or 'Dilate' operation. Second, the 'Active Contours' algorithm was applied for better matching with SR101-labeled signals. Finally, the 'Smooth Edges' algorithm was applied to smooth the mask in 3D space. The constructed mask was then saved to Matlab workspace for further use.

### X/Y-axis projection and time-course trajectory of signal-weighted centroid

A 40 μm-cubic area was cropped centered at astrocytic soma from the original field of view. The mean projection to X or Y axis by formula (1). Center weighted by calcium signal intensity was calculated by formula (2). The trajectory was sequenced by a series of weighted centroid coordinates. Finally, the trajectory was converted to a volumetric image stack for visualization by mapping coordinates to pixels.

1 $ \mathrm{P}\mathrm{r}\mathrm{o}\mathrm{j}\mathrm{e}\mathrm{c}\mathrm{t}\mathrm{i}\mathrm{o}\mathrm{n}\left(\mathrm{x}\right)=\mathrm{m}\mathrm{e}\mathrm{a}\mathrm{n}\left[\mathrm{m}\mathrm{e}\mathrm{a}\mathrm{n}\left(3\mathrm{D}\;\mathrm{m}\mathrm{a}\mathrm{t}\mathrm{r}\mathrm{i}\mathrm{x},\mathrm{y}\right),\mathrm{z}\right] $

2 $ {C}_{i}=sum\left\{\frac{\left(1:\mathrm{m}\right).\mathrm{*}\mathrm{P}\mathrm{r}\mathrm{o}\mathrm{j}\mathrm{e}\mathrm{c}\mathrm{t}\mathrm{i}\mathrm{o}\mathrm{n}\left(\mathrm{i}\right)}{\mathrm{s}\mathrm{u}\mathrm{m}\left[\mathrm{P}\mathrm{r}\mathrm{o}\mathrm{j}\mathrm{e}\mathrm{c}\mathrm{t}\mathrm{i}\mathrm{o}\mathrm{n}\right(\mathrm{i}\left)\right]}\right\}$

*C_i_*, center along i, where i in (x,y,z), *=elementwise multiplication

### Signal distribution over distance-to-center

First, the soma and main branch mask were created by Volume Segmenter App. Next, the center of mask is calculated by formula (3). Euclidean distance to the mask center was calculated and assigned to each pixel. Finally, the mean pixel intensities at each distance were calculated by grouping and averaging on distance.

3 $ {D}_{i}=\frac{(1:\mathrm{n}).\mathrm{*}\mathrm{P}\mathrm{r}\mathrm{o}\mathrm{j}\mathrm{e}\mathrm{c}\mathrm{t}\mathrm{i}\mathrm{o}\mathrm{n}\left(\mathrm{i}\right)}{\mathrm{s}\mathrm{u}\mathrm{m}[\left(1:\mathrm{n}\right).\mathrm{*}\mathrm{P}\mathrm{r}\mathrm{o}\mathrm{j}\mathrm{e}\mathrm{c}\mathrm{t}\mathrm{i}\mathrm{o}\mathrm{n}(\mathrm{i}\left)\right]} $

*D_i_*, center of mask along i, where i in (x,y,z), *=elementwise multiplication

## Results

The overall goal of this study is to establish a pipeline for 3D volumetric time-lapse images and visualize the spatiotemporal distribution of volumetric signals. Furthermore, we utilized the pipeline to compare astrocytic calcium signals under quiet wakefulness and isoflurane anesthesia.

### Time projection of volumetric time-lapse astrocytic Ca^2+^ signals

**Figure 1:**
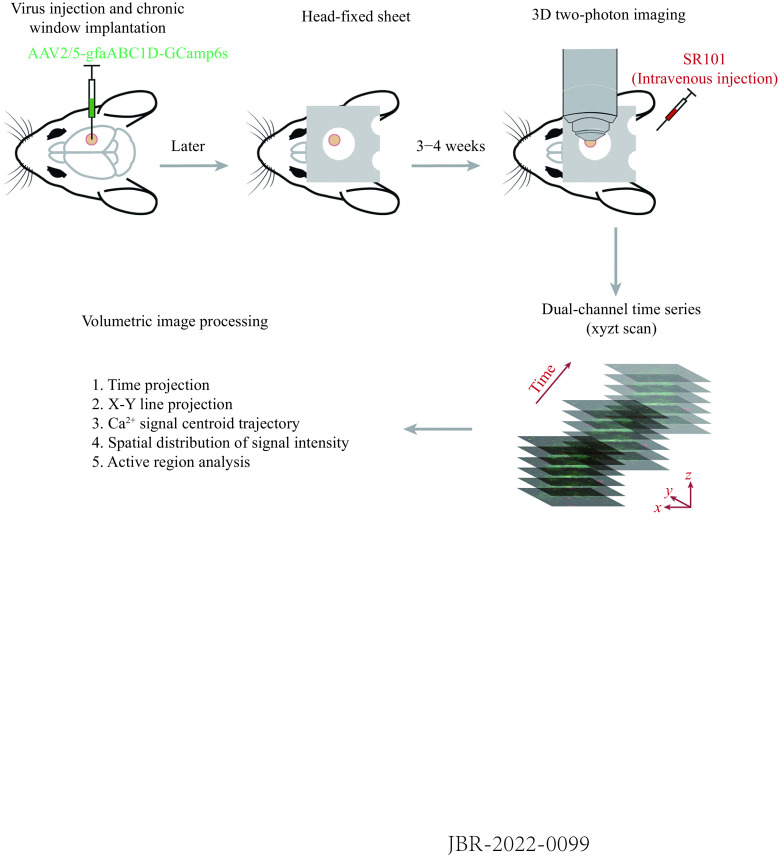
Imaging paradigm and analysis procedure.

**Figure 2:**
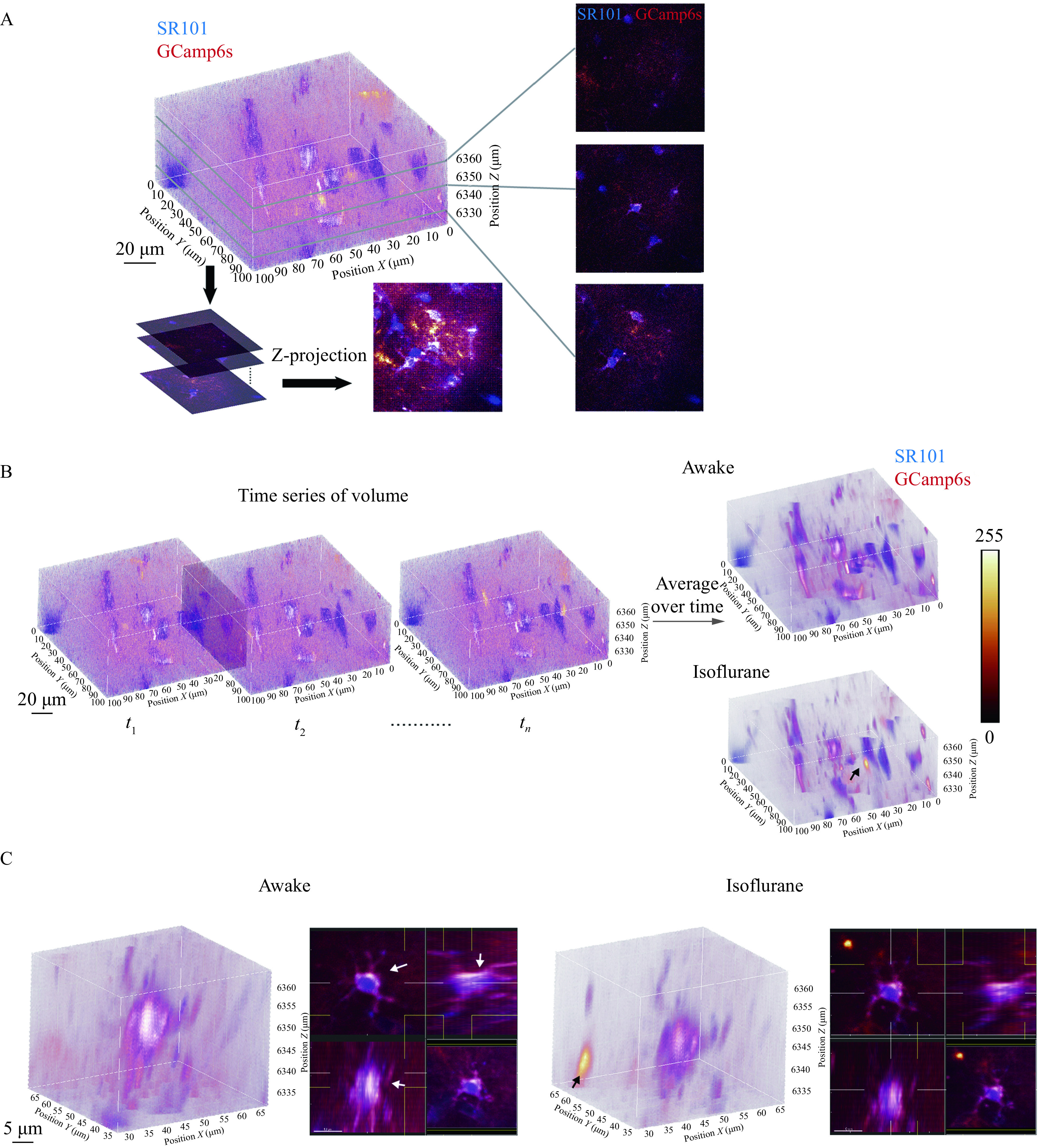
Volumetric image visualization of awake and anesthetized astrocytic Ca^2+^ signals.

About 2 to 3 minutes of 100 μm × 100 μm × 50 μm volume were sampled during quiet wakefulness and isoflurane anesthesia (0.1 L/minute, 1.5% isoflurane in air, ***Fig. 1***). We obtained three representative layers from the volume to compare with z-projection of the whole volume (***Fig. 2A***). The z-projection merged signals from the extra vertical dimension (z-axis) captures more enriched events of astrocytic activies than any of the three single layers, demonstrating that the volumetric imaging was able to capture previously undetected signals by 2D scanning.

Time projection of volume series generated a heatmap of Ca^2+^ signals illustrated the time-course accumulation of astrocytic activities, or functional hotspots. The original volume covered the soma of 6 to 7 SR101-labeled astrocytes; thus, the paradigm was able to capture the signal dispensation in both proximal and distal region. We found an overall lower brightness of the averaged Ca^2+^ signal in the heatmap both inside and outside of SR101-labeled region (***Fig. 2B***). We also observed a distal-to-soma hotspot of a 5 to 10 μm radius in anesthetized state (black arrow, ***Fig. 2B*** and ***C***) rather than in wakefulness. To visualize the Ca^2+^ signals in the soma and main branch region, we cropped a 40 μm cubic volume out of the original image stack. The extended 3-way (XY, YZ and XZ) projections showed that the most active area laid in the shell-shaped component within cytoplasm. Among the active component, an ovary-shaped region extended along the z-axis was in a persistent active state (white arrow, ***Fig. 2C***), suggesting a housekeeping functional hotspot.

**Figure 3:**
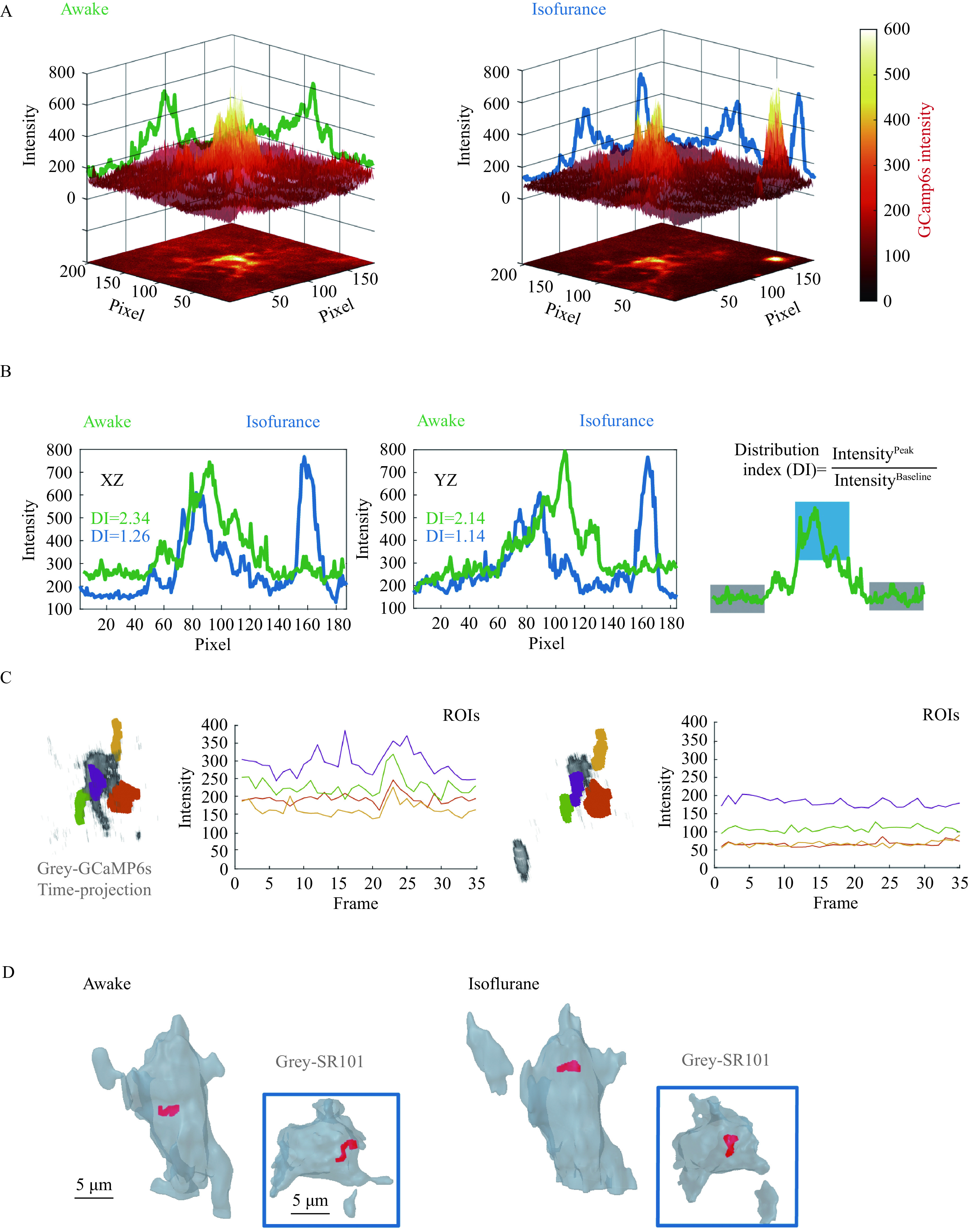
Spatiotemporal distribution of astrocytic Ca^2+^ signals.

Next, we projected the 3D heatmap stack to XY plane and plotted the max intensity value over x-axis and y-axis, respectively (***Fig. 3A***). The height of the projected heatmap coded signal intensity. As observed in volumetric illustration, we found the pixel intensity level was generally lower in anesthetized state (darker on heatmap). The projected curve illustrated the intensity distribution along the axis (***Fig. 3B***, left). To quantify the distribution balance between distal and proximal components, distribution index was introduced as the ratio of peak to baseline on the curve (***Fig. 3B***, right). The Ca^2+^ signals concentrated more within soma during wakefulness.

### Time-course weighted trajectory of astrocytic Ca^2+^ signals

The time projection heatmap preserved the cumulative activity that indicated functional hotspots. However, the operation discarded the frame-by-frame dynamic information. To illustrate the temporal fluctuation of Ca^2+^ signaling, we first selected several manually labeled volumes of interest by masking. The pixel intensities in the masked regions were plotted over time frames (***Fig. 3C***). The signal traces in anesthetized condition represented lower intensity level and less fluctuation, implying seized astrocytic activities. In order to integrate the dynamic moving feature, we next calculated the time-course trajectory of Ca^2+^ signal-weighted centroid. The centroid demonstrated the central tendency of Ca^2+^ signals and concentrated the spatial distribution into one set of coordination. The frame-by-frame centroids formed a trajectory illustrating the movement of astrocytic Ca^2+^ center. The trajectory was mapped back to the original volumetric space and overlapped onto a 3D reconstructed surface based-on SR101 labeling (***Fig. 3D***). We found both awake and anesthetic Ca^2+^ centroid moved in a small restricted regions with similar sizes. The awake trajectory was closer to the functional hotspot from time projection heatmap, suggesting a more concentrated distribution of Ca^2+^ signals than that of anesthesia.

### Distribution of Ca^2+^ signals inside and distal to SR101-labeled region

**Figure 4:**
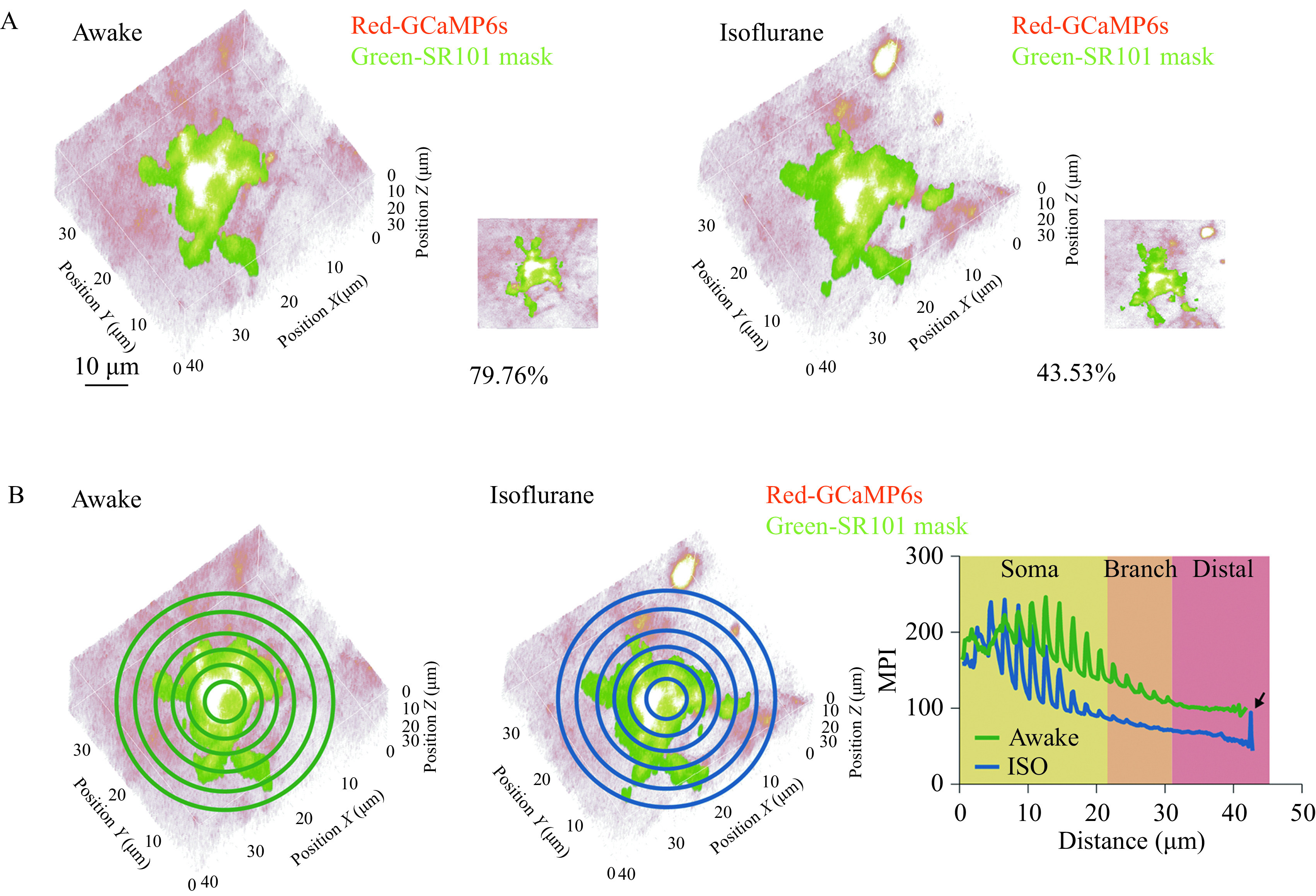
Ca^2+^ signal distribution in and outside of SR101-labeled region.

Considering the differently distributed Ca^2+^ signals under wakefulness and anesthesia, we next quantified the active regions inside and outside the region of soma and main branches. For the inside part, the percentage of active volume was calculated within an SR101-masked region. We first semi-manually constructed an SR101-positive mask. Then, the percentage of Ca^2+^ signals within the mask above the threshold of lower than 25% quantile was computed. We found a substantial lower ratio of active spots under isoflurane anesthesia (43.53% *vs.* 79.76%, ***Fig. 4A***). To summarize the Ca^2+^ signal around the soma and main branch region, we plotted the mean pixel intensity (MPI) versus the distance from the center of the SR101 mask (estimated center of soma). On the curve, we found a decreasing baseline with regular peaks within soma range showing that the active spots arranged proximally. Isoflurane anesthesia showed a generally lower Ca^2+^ signal intensity, but the peaks on the curve at distal region indicating remote hotspots were associated with an anesthetic state (***Fig. 4B***, black arrow).

## Discussion

In this study, we established a pipeline for imaging 4D time-lapse volumetric data *in vivo* and visualizing spatiotemporal patterns of astrocytic Ca^2+^ signals. The pipeline was utilized to compare astrocytic activities under quiet wakefulness and isoflurane anesthesia. We found astrocytes under anesthesia exhibited lower overall Ca^2+^ activities, smaller active regions, and unique functional hotspots distal to soma.

### Advantages and potential applications of the visualization pipeline

Accumulating evidence has pointed to the idea that astrocytes integrate brain state with local neuronal and vascular activities^[26]^. Calcium signals, as a main readout of astrocytic function, are shown to mediate the integration. The spatial information loss by conventional 2D imaging may bias the understanding of astrocytic functional integration, while 3D Ca^2+^ imaging of astrocytes adds another dimension of information, as well as another level of complexity. The 3D Ca^2+^ imaging of astrocytes requires the development of next-generation data-analysis tools to evaluate dynamic changes in the volume and the progress of Ca^2+^ events. Volterra *et al* developed a set of analytical tools for volumetric imaging correction and functional parcellation by seed-based correlation^[26]^, but there still lack tools illustrating hotspots and extracting functional connectivity among subdomains. As voxel-wise description and hotspot detection are basic steps of deeper analysis and understanding of the 3D spatial integration, we developed this pipeline. To better demonstrate the ability of the tool, we compared two-photon astrocytic calcium imaging under wakefulness and isoflurane anesthesia. Anesthesia is a readily induced and stably altered brain state, which profoundly modifies astrocytic Ca^2+^ signals^[23]^, cortical neuronal activities^[27]^, vascular dynamics^[28]^ and brain connectivity^[29]^. Therefore, anesthetic condition by isoflurane provides a model for studying information integration by astrocytes. Functional hotspots revealed state-dependent subdomains, while weighted-center trajectory exhibited general fluctuation level in two states. A direct expansion of application could be detecting hotspots and signal trajectory under various behavioral states. Potentially, our visualization technique provides more information than conventional 2D imaging for studying heterogenicity of astrocytes physiology as it maps calcium signals from the whole cellular territory. Furthermore, neuron-astrocyte interaction subdomain study could be visualized by combining the pipeline with optogenetically manipulated neuronal axons.

### Limitations and disadvantages of the visualization pipeline

Genetically encoded calcium indicator GCamp6s is used to measure astrocytic Ca^2+^ transients, which increases its fluorescence intensity when binds to Ca^2+[30]^. It is widely used for its good combination efficiency and long-term stable expression. However, it has been shown that GCamp6s cannot fully characterize intracellular Ca^2+^ changes according to its binding level. SR101 is a widely used, efficient and specific dye for calibrating astrocyte volume. The limitations include time-dependence and neuronal toxicity. Cells cannot be accurated calibrated beyond 4 hours due to catabolism. Moreover, when incubated in a concentration over 100 μM, SR101 induces cortical seizure-like activity^[31]^. In addition, GCamp6s and SR101 show expression heterogeneity in different brain regions. The shortcomings are to be overcome with more efficient AAV-based morphological and functional double labeling.

With the development of resonant-metric scan mirrors, two-photon microscopy can scan at an adequately high speed to capture major events of astrocytic Ca^2+^ activities. However, the slow speed in the third dimension (the z-axis) limits the overall sampling rate. To cover the whole astrocytic domain, researchers must balance between reduced z-axis resolution and sampling rate. Thus, the rapid Ca^2+^ manifestations faster than sampling rate in z-axis might go undetected. To make up this flaw, the recently developed Z-Axis Piezo Scanning Stage enables faster z-axis, resulting in a better sampling rate.

Astrocytes are a highly diversified cell population. The heterogeneity of astrocytes has drawn significant attention^[32]^. As we have discussed, a heterogeneity study may benefit from 3D imaging, but the diversified morphology produces an obstacle for inter-cellular comparison, as there is currently no formalized morphology template for subtypes of astrocytes. The relatively shallow imaging depth of two-photo microscopy also limits the study of heterogeneity as cortical astrocytes exhibit highly layer-dependent features^[33]^.

### Future 3D event-based, decomposition and network functional analysis

Currently, no single algorithm or toolbox can capture the broader scale diversity of Ca^2+^ signals in astrocytes. Fortunately, decades of development in human brain functional imaging provides plenty of tools to extract relevant biological insights from volumetric imaging. Like human functional brain imaging, cellular volumetric functional imaging presents time-lapse 3D volumetric data with a sampling rate of hundreds of milliseconds to seconds. The combination and translation of several existing analytical techniques and ideas would offer a set of strategies that can extract significant information from 3D Ca^2+^ transients.

Astrocytic domains are believed to be constituted by a range of functional subdomains. Conventional 2D XYT imaging loses up to 80% information^[24]^, while volumetric imaging provides full spectral of information and leaves the task to be solved computationally. In this study, we calculated metrics in the scope of a whole cell. To better capture biologically insightful processes, events detection are of great importance. An event includes astrocytic Ca^2+^ activity channel pixels that are often clustered into small groups by the spatial neighboring and temporal continuity. Event detection algorithms are often utilized interchangeably with automatic functional domain segmentation, as both techniques aim at detecting functional spatial units. Novel techniques for this purpose are emerging, including CNMF-based method^[34]^, CALIMA^[35]^, CaImAn^[36]^, and AQuA^[37]^.

This study aimed at demonstrating activity-related signal distribution and providing a pipeline to visualize the spatiotemporal features. However, more intrinsic and stochastic patterns hidden behind the visualized intuitive pattern are yet to discover. Network modeling based on graph theory is a great model for studying dynamic relationship of functional related units. Astrocyte volumetric images could be converted into a graph by different correlation metrics among functional subdomains^[38]^. Spatio-Temporal Tensor Analysis and Spatio-Temporal Representation Factorization and other DNN-based techniques (deep neural network) provide a computational framework for feature extraction^[39–40]^. In summary, an expansion of event detection, decomposition method, and network analysis into 3D space could enable a vast progress in astrocytic functional research.
